# Breastfeeding: science and knowledge in pediatric obesity prevention

**DOI:** 10.3389/fmed.2024.1430395

**Published:** 2024-09-27

**Authors:** Marilena Muraglia, Maria Felicia Faienza, Roberta Tardugno, Maria Lisa Clodoveo, Carmen Matias De la Cruz, Fátima German Bermúdez, María Gabriela Munizaga, Luz Valencia, Filomena Corbo, Andrea Orellana-Manzano

**Affiliations:** ^1^Department of Pharmacy - Drug Science, University of Bari ‘Aldo Moro’, Bari, Italy; ^2^Pediatric Unit, Department of Precision and Regenerative Medicine and Ionian Area, University of Bari “A. Moro”, Bari, Italy; ^3^Interdisciplinary Department of Medicine, School of Medicine, University of Bari-Aldo Moro, Bari, Italy; ^4^Laboratorio Para Investigaciones Biomédicas, Facultad de Ciencias de la Vida, Escuela Superior Politécnica del Litoral, ESPOL, Guayaquil, Ecuador; ^5^Licenciatura en Nutrición y Dietética, Facultad de Ciencias de la Vida, Escuela Superior Politécnica del Litoral, ESPOL, Guayaquil, Ecuador

**Keywords:** pediatric obesity, breastfeeding, epigenetic, Mediterranean diet on breastfeeding, nutrition

## Abstract

The increasing prevalence of childhood obesity worldwide is a significant concern due to its link to severe health issues in adulthood, such as non-communicable diseases (NCDs). To address this issue, this review evaluates the effectiveness of various preventive measures for childhood obesity, focusing on maternal nutrition and breastfeeding. The study underscores the criticality of the periconceptional period, where the diets of both parents can influence epigenetic modifications that impact the child’s metabolic pathways and obesity risks. Breastfeeding is a potent protective mechanism against early-onset obesity, significantly enhancing the infant’s metabolic and immune health by modifying DNA methylation and gene expression. Furthermore, the perspective underscores the significance of the Mediterranean diet during the periconceptional period and lactation. This diet can effectively prevent gestational complications and improve breast milk quality, fostering optimal infant development. Recognizing that obesity results from genetic, epigenetic, environmental, and social factors, the paper advocates for a comprehensive, multidisciplinary approach from the earliest stages of life. This approach champions a balanced maternal diet, exclusive breastfeeding, and timely introduction to complementary foods. In conclusion, addressing pediatric obesity requires a multifaceted strategy emphasizing improving prenatal and postnatal nutrition. Further research is necessary to understand the epigenetic mechanisms influenced by nutrition and their long-term effects on children’s health. This will help refine interventions that curb the obesity epidemic among future generations.

## Introduction

The World Health Organization (WHO) has key strategic goals for preventing and treating pediatric obesity and its complications. The report “Prevalence of obesity among adults BMI ≥ 30, crude Estimates” prepared by WHO in 2022 shows that 18% of children and adolescents globally are overweight and obese (1).

In particular, the number of children with obesity has increased tenfold: from 5 and 6 million in 1975 to 50 and 74 million in 2016 for boys and girls, respectively. An analysis of data collected after 2000 predicts that obesity will affect 25 percent of all children under the age of 16 by the end of 2050 (2, 3).

The significance and relevance of these data led WHO to define obesity as a global public health problem. The reasons for this emergence lie essentially in the fact that being overweight and obese, in parallel with low physical activity, are among the main factors responsible for the onset of disabling and noncommunicable diseases (Non-Communicable Diseases-NCDs), among which are ischemic heart disease, stroke, hypertension, type 2 diabetes mellitus, and osteoarthritis, the leading causes of death globally (1).

Numerous studies link the existence of genes in the genome to obesity; nevertheless, these genes can be expressed differently because of environmental influences. It is essential to create ideal eating conditions for the child to develop during this stage and even while breastfeeding. The mother’s eating habits during conception and the periconceptional period are closely related to the triggering of specific patterns that direct the newborn to develop it during its early childhood and later adulthood (4, 5).

## Epigenetic implications of maternal and paternal nutrition during the periconceptional period

The periconceptional period is critical for epigenetic programming and childhood obesity risk (6). Maternal/paternal diet and obesity lead to epigenetic changes that affect the child’s metabolism and body fat regulation (7–9). A low-fat diet can reverse these epigenetic modifications (10).

The periconceptional period, which encompasses the weeks before and after conception, is crucial for epigenetic programming (11, 12). The developing embryo is highly susceptible to environmental influences during this critical window. Epigenetics involves changes in gene expression that do not alter the DNA sequence itself but modify how genes are turned on or off (13). These epigenetic changes, influenced by diet, lifestyle, and environmental exposures during the periconceptional period, can have long-lasting effects on an individual’s health and development (13–16). Several studies show that maternal nutrition shapes the fetal epigenetic landscape, with poor diets and maternal obesity causing epigenetic modifications that affect the offspring’s metabolism, adipogenesis, and appetite regulation (17–20). High-fat maternal diets alter DNA methylation, increasing the risk of childhood obesity (21, 22). In addition, paternal factors such as obesity and diet influence epigenetic programming through sperm (23).

In consequence, maternal nutrition during the periconceptional period, defined as the timeframe of approximately 5-6 months before and after conception, plays a crucial role in reproductive health and the long-term well-being of offspring. This period is sensitive to various nutritional influences, including deficiencies or excesses in essential nutrients. For instance, maternal diets lacking in methyl donors like folate and vitamin B12 can lead to global hypomethylation in fetal DNA, potentially increasing the risk of developmental disorders and chronic diseases later in life (24). Conversely, maternal overnutrition, often linked to obesity, can adversely affect oocyte quality and embryonic development, resulting in smaller oocytes and altered mitochondrial function, possibly leading to increased inflammatory responses in the embryo (25). Research indicates that the quality of maternal diet during this critical window not only influences immediate reproductive outcomes but also sets the stage for the health trajectories of the offspring, affecting their susceptibility to conditions such as obesity, cardiovascular diseases, and metabolic disorders in adulthood (7, 26). For this, maternal obesity has been shown to induce significant epigenetic changes that can predispose offspring to develop insulin resistance and other metabolic disorders later in life. Research indicates maternal obesity alters placental gene expression and leads to epigenetic modifications, such as changes in DNA methylation and histone modifications, which can affect the offspring’s ng’s phenotype (17). For instance, studies have demonstrated that a maternal high-fat diet can increase the methylation of genes associated with metabolic pathways, leading to an upregulation of lipogenic processes in the offspring. This epigenetic reprogramming can create a lasting impact, predisposing children to obesity and cardiometabolic diseases as they grow older (27). The mechanisms underlying these epigenetic changes include a disrupted intrauterine environment that affects fetal development and metabolic programming. Maternal obesity leads to chronic low-grade inflammation and altered nutrient availability, which together influence the fetal epigenome (28). For example, specific studies have identified DNA methylation changes in genes linked to fatty acid metabolism and mitochondrial function in children born to obese mothers. These alterations are not only immediate but can persist into later stages of development, highlighting the critical role of maternal metabolic health during pregnancy in shaping the long-term health outcomes of offspring.

Specific epigenetic changes observed in the offspring of obese mothers include alterations in DNA methylation and histone modifications, which can affect the expression of genes related to metabolism and glucose regulation (17). DNA methylation is one of the most studied epigenetic mechanisms; research has shown that children of obese mother’s exhibit altered methylation patterns in genes that regulate lipid metabolism and insulin sensitivity. For example, studies have demonstrated that exposure to an obesogenic maternal environment can lead to hypermethylation of certain genes crucial for weight control and glucose homeostasis, thereby predisposing offspring to develop insulin resistance and obesity in adulthood (17). Additionally, histone modifications also play an important role in the epigenetic programming of children born to obese mothers. These modifications can influence chromatin structure and, consequently, the accessibility of DNA to transcription factors, affecting gene expression (29). Research has indicated that epigenetic changes can be inherited and persist across generations, suggesting that maternal obesity not only impacts the immediate health of the child but may also have transgenerational effects (30).

The results of the relationship between H19DMR methylation with obesity and the expression of the H19 and IGF2 genes indicate that fat intake during the first trimester of pregnancy significantly impacts DNA methylation in the IGF2 and H19 genes in newborns. Higher consumption of omega-6 polyunsaturated fats was associated with lower IGF2-DMR methylation and higher H19-DMR methylation. On the other hand, an increased intake of omega-3 polyunsaturated fats was associated with decreased H19-DMR methylation. These findings suggest that maternal diet in early pregnancy may influence DNA methylation, affecting fetal development and long-term health. These findings underscore the importance of maternal nutrition and metabolic status during pregnancy, as interventions during this critical period could help mitigate the risks of metabolic diseases in offspring (31).

Paternal nutrition plays a crucial role in periconceptional health, influencing both male fertility and the health of future offspring (23). Adequate nutritional status in men before conception is associated with improved sperm quality, including sperm count, motility, and morphology. Nutrients such as folate, zinc, and antioxidants are vital to produce healthy sperm (32). Deficiencies in these nutrients can lead to impaired reproductive function and may increase the risk of genetic abnormalities in sperm, which can affect the health of the child. Moreover, paternal nutrition can have long-term implications for the health of the offspring. Research indicates that the nutritional environment experienced by the father can influence epigenetic changes in sperm (23), which may affect fetal development and the child’s health later in life. For instance, a diet high in saturated fats and low in fruits and vegetables has been linked to adverse health outcomes in children, such as obesity and metabolic disorders (14).

Considering this evidence, body mass index (BMI) at conception, diet, lifestyle, and maternal weight gain are recognized as metabolic modulators of obesity in the newborn. Among all the interventions promoted in a multifactorial strategy, breastfeeding of infants has been recognized as the most essential method, in the first 1,000 days of life, to reduce the significant risk factors recognized for the development of pediatric obesity. Analysis of statistical data derived from clinical trials in the field shows that each additional month of breastfeeding compared with the reference sample results in a reduction in the prevalence of pediatric obesity of 4% (33, 34).

Some authors investigated the effects of male body mass index (BMI) on sperm DNA methylation and its association with next-generation fetal cord blood (FCB) DNA methylation. The results suggest that male obesity is nominally associated with sperm DNA methylome modifications, which could affect the next-generation epigenome (35). However, more research is needed to confirm these findings. So, this highlights the importance of not only focusing on maternal nutrition but also emphasizing the role of fathers in contributing to a healthy gestational environment through their dietary choices.

In addition to these points, studies have shown that paternal obesity and metabolic health can significantly impact offspring health (14). Research indicates that fathers with obesity are more likely to have children with increased risks of obesity, diabetes, and cardiovascular diseases later in life. Furthermore, a systematic review highlighted that paternal lifestyle factors, including diet and physical activity, are associated with the risk of developing metabolic syndrome in their children. Likewise, this review highlights, that obesity in men negatively affects sperm quality and offspring health, demonstrating intergenerational epigenetic effects related to nutritional and lifestyle factors. Obesity is associated with alterations in DNA methylation and sperm molecular characteristics, which may influence pregnancy success and offspring health. To address these issues, the concept of Paternal Origins of Health and Disease (POHaD) is proposed (36).

Another study found that paternal obesity is significantly associated with hypomethylation at the IGF2 differentially methylated region (DMR), with a b-coefficient of -5.28 (*p* = 0.003), indicating a notable impact on the methylation levels in newborns. In contrast, no significant associations were observed at the H19 DMR. These findings suggest that paternal lifestyle factors, particularly obesity, may disrupt normal genomic imprinting processes, potentially influencing the health of future generations. Also, this study analyzed the relationship between paternal body mass index (BMI) during the periconceptional period and DNA methylation in the blood of newborns. Paternal BMI of 25 or more was found to be associated with higher birth weight and significant changes in DNA methylation at 9 CpG sites, independent of maternal BMI. Hypomethylation at site cg04763273 decreased by 5% with each 1-unit increase in paternal BMI and was maintained at 3 and 7 years. In addition, methylation at site cg01029450 in the promoter region of the ARFGAP3 gene was also associated with lower birth weight and higher BMI z-score at 3 years. These findings suggest that paternal BMI may influence offspring’s epigenetic health and development (37). These findings underscore the need for public health initiatives that promote healthy dietary practices among men of reproductive age, aiming to improve not only their health but also the future health of their children.

The composition of breast milk is critical for the healthy development of newborns, and its variability can be influenced by maternal factors, such as body mass index (BMI) and obesity (38). Recent studies have shown that overweight or obese mothers may have breast milk with altered metabolic profiles, which could affect the long-term health of their infants (39).

A meta-analysis indicated that maternal obesity is correlated with an increased risk of childhood overweight, suggesting that breast milk may play a crucial role in this relationship (40). Furthermore, another research has shown that breast milk from obese mothers has significant differences in the concentration of metabolites, such as amino acids and fatty acids, compared to normal-weight mothers, which may influence infant body composition and postnatal growth. Metabolomics, a technique that studies metabolite profiling in biofluids, has been used to investigate these differences in breast milk (41). For example, metabolites such as fatty acids and oligosaccharides may be altered in the milk of mothers with elevated BMI, which could have implications for the metabolic health of infants. These findings suggest that assessment of breast milk composition about maternal BMI is crucial to better understanding how nutrition early in life may influence metabolic disease risk in infancy and later in life (42).

## Alteration in the methylation of genes related to obesity and breastfeeding

Breastfeeding is a crucial factor in the healthy development of infants, and its influence on the genome can have long-lasting effects on health and well-being. Genetic alterations in breastfed infants may positively influence their stress response, allowing their bodies to adapt more effectively to stressful situations (43). In addition, breast milk may modify the effect of genetic profiles related to conditions such as asthma, suggesting a protective effect in infants at genetic risk (44). This may translate into a reduced risk of diseases ranging from allergies and infections to digestive and respiratory problems (45, 46). Finally, it significantly impacts the methylation of metabolism-related genes and the immune system (47). The absence of breastfeeding has been associated with increased DNA methylation. In other words, it can influence DNA methylation, an epigenetic process involving adding methyl groups to DNA nucleotides (48, 49). DNA methylation can affect gene expression, silencing or activating gene transcription. In addition, studies have shown that DNA methylation can affect the expression of genes related to long-term infant metabolism, such as the leptin gene, which affects the regulation of metabolism and storage (50–52). DNA methylation in specific genes is associated with fat metabolism and inflammation, increasing the risk of metabolic disorders and obesity later in life (47, 53).

One of the key mechanisms of nutritional programming during the postnatal period is through epigenetic modifications. Nutrition in early life, especially during breastfeeding, can have a significant impact on the child’s gene expression through epigenetic changes such as DNA methylation. Several studies have explored the link between breastfeeding and epigenetic programming related to obesity and metabolism (54).

One study found that breastfeeding can induce epigenetic effects on genes associated with obesity in children as young as one year of age (55, 56). Another interesting finding is the association between the duration of breastfeeding and DNA methylation in genes related to metabolism and appetite control. As this article shows, the duration of breastfeeding affects DNA methylation in genes related to metabolism and appetite control in one-year-old infants. Through the MANOE study, 101 mother-infant pairs were analyzed, finding that longer duration of breastfeeding is associated with significant epigenetic changes in the RXRA and LEP genes, which may influence the development of childhood obesity. The results suggest that breastfeeding not only impacts physical growth but also metabolic regulation through epigenetic modifications (54).

The recommendation of exclusive breastfeeding for the first 6 months of life is supported by numerous studies demonstrating its benefits for infant health. However, evidence suggests that extending breastfeeding beyond this period may offer additional protection against obesity. Breastfeeding was associated with a 13% reduction in the likelihood of overweight or obesity (57). In addition, a longitudinal study showed that each additional month of exclusive breastfeeding was associated with a 1% decrease in body mass index (BMI) and a 2% reduction in fat mass at 6 years of age (58). These findings underscore the importance of prolonged breastfeeding as an obesity prevention strategy. The mechanism by which prolonged breastfeeding and prolonged suckling contribute to obesity risk reduction is multifaceted. First, the unique composition of breast milk, which changes over the time of breastfeeding, provides bioactive factors that influence the metabolic development of the infant (59).

On the other hand, prolonged sucking may play a crucial role in the self-regulation of intake. A study of breastfed babies showed a better ability to regulate their food intake compared to bottle-fed infants (60). This could be because breastfeeding allows the infant to control milk flow and feeding duration, which may promote the development of more effective satiety mechanisms. In addition, prolonged sucking may influence orofacial and jaw muscle development, which could have implications for later feeding and chewing patterns (61).

## Multifactorial development of childhood obesity linked to cardiometabolic risks

**Figure 1:**
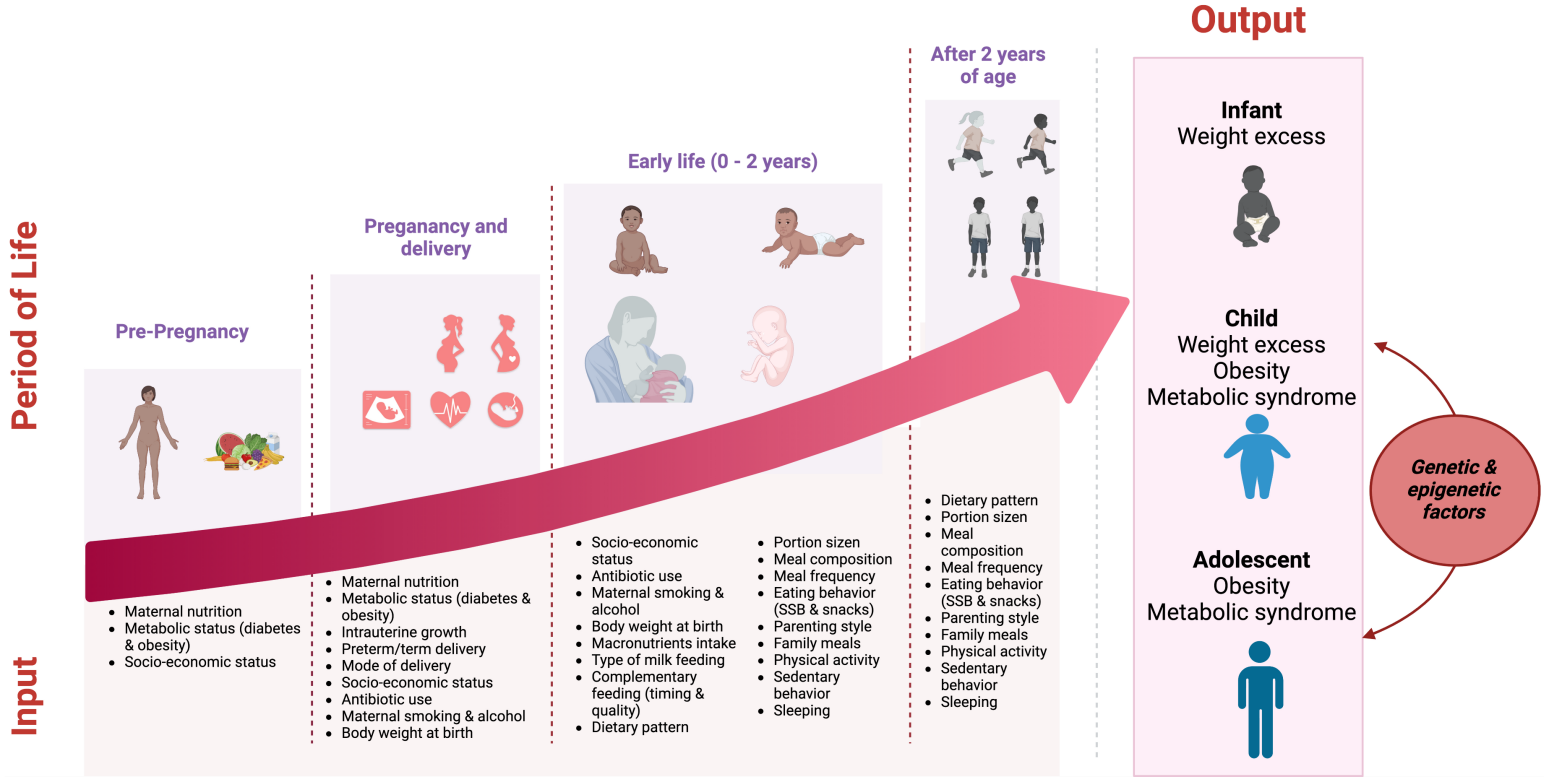
Multifactorial development of childhood obesity.

Reducing childhood and adolescent obesity should be approached from a multifactorial and multidisciplinary perspective, encouraging the promotion of healthy behaviors from the first months of life, breastfeeding, and adequate nutrition of the child and the family, and interventions capable of modifying biological, environmental, and social determinants can be implemented at multiple levels (Figure 1).

Obesity is not simply a multisystem disease involving systems and organs. Instead, it is a complex, multifactorial condition resulting from the interaction of biological (genetic and epigenetic), psychological, and environmental factors, often accompanied by psychosocial (low self-esteem, body image rejection) and relational (stigma and bullying) factors that lead to increasingly common complications in obese children/adolescents. All these factors result in the accumulation of adipose tissue, mainly in the abdominal area, associated with cardiometabolic complications that tend to increase in adulthood as obesity persists (62).

However, it would be a very serious mistake to think that diet and physical activity are the only factors that are resistant to the risk of developing NCDs. Considering, therefore, the consequences, both in terms of morbidity-mortality and socioeconomic, that obesity determines, a program of interventions to encourage prevention, information, and treatment of the disease is urgently needed. All preventive measures aim to combat the phenomenon of childhood obesity at an early stage to prevent its consequences in adulthood (62, 63).

## Benefits of breastfeeding in the prevention of childhood and adult obesity

One strategy to prevent the risk of obesity in adulthood is exclusive breastfeeding during the first six months of life, as recommended by the WHO (63). It also suggests that breastfeeding should continue in the adequate presence of complementary baby foods until at least the second year. Statistical evidence highlights the correlation between pediatric weight gain and early cessation of breastfeeding. For these children, breastfeeding is important because it has been shown that in the first 7-12 months of life, it increases the likelihood that their weight will be proportional to their development; in contrast, infants fed with artificial milk are overweight. Studies show that formula milk does not adjust to the caloric needs of the infant, increasing the significant risk of obesity and cardiovascular disease in children, especially in those who are above gestational age and average birth weight (33).

Therefore, the preventive action of breastfeeding on the risk of obesity in both children and adults is attributable to the role played by the bioactive components of breast milk in the correct and harmonious immunological, endocrinological, neurological, and physiological child’s growth. Unlike breast milk, the macronutrient composition of infant formulas has a higher amount of proteins that can alter the child’s growth curve, doubling the risk of overweight and obesity compared to breastfed infants. Infant formula, proposed as an alternative to breast milk, causes a higher insulinemic spike than breast milk, resulting in increased fat accumulation and adipose mass with consequent risk of pediatric obesity (34). Indeed, breast milk is a complex food with numerous bioactive components that play a crucial role in infant development and metabolic programming (64). Leptin has been the subject of much attention in breastfeeding and obesity research (64).

Leptin present in breast milk is considered a key factor in the regulation of appetite and energy balance in infants. Studies have shown that leptin levels in breast milk are inversely related to infant weight gain and adiposity in the first months of life (65). In addition, it has been observed that breastfed infants have a greater ability to self-regulate their food intake compared to formula-fed infants, which could be related to early exposure to leptin (66).

Other bioactive components of breast milk, such as adiponectin, ghrelin, and insulin-like growth factors (IGFs), also appear to play important roles in metabolic programming. For example, adiponectin in breast milk is associated with a lower risk of childhood overweight (67). Exposure to these factors during lactation may influence adipose tissue development, insulin sensitivity, and appetite control mechanisms, potentially establishing metabolic “programming” that may affect the risk of obesity and metabolic disease later in life (68).

In addition, breastfed babies are more likely to have a varied and healthy diet, consuming more fruits and vegetables than formula-fed babies (69). Breast milk also influences the infant’s gut microbiota, which is found to be particularly rich in microorganisms than in obese infants. Microbial colonization, which has already begun in the intrauterine phase, continues during skin-to-skin contact immediately after birth, with the first feedings of colostrum, and with breastfeeding. Thus, the mother is the major microbial source of infants. Although the mechanism by which the maternal microbiome influences the development of the baby’s microbiome is unclear, it is scientifically certain that the colonization process and the development of the gut microbiota positively affect metabolic and immune development (70, 71).

Breast milk is the main factor that constitutes the intestinal microbiota (72), however, it can be highlighted, concerning studies carried out, that this bacterial profile in the infant can be affected by the mother’s diet, representing an important etiological factor (73). It has been demonstrated that variables related to nutritional status have an influence on the composition of the intestinal microbiota, showing that pathological conditions such as obesity and high-fat mass can consequently generate intestinal dysbiosis. Newborns shape their intestinal microbiota according to different factors such as type of delivery, gestational age of the mother, initial feeding, and exposure to external agents (73, 74). The microbiota of infants is mostly composed of enterobacteria (*Escherichia coli or Klebsiella pneumoniae*) (75–77).

A study sought to differentiate the composition of the intestinal microbiota of obese patients from lean patients, recording that there was a higher population of Firmicutes/Bacteroidetes in obese patients, whose main characteristic is to extract more energy from food and generate weight gain. It was also identified that in obese patients there was a greater presence of Escherichia and *Eschericha albertii*, which were identified as biomarkers of obesity (78).

It should be emphasized that weight loss is not the main factor modifying the composition of the intestinal microbiota, but also the type of diet maintained by everyone. Scientific evidence shows that, if exclusive breastfeeding is maintained until 6 months of age, before the introduction of complementary feeding, there is a greater predisposition to the colonization of bifidobacteria compared to formula-fed infants (79–81). Likewise, once the introduction of solids is initiated and the percentage of breast milk consumption decreases, there is an important change in microbial diversity, since bacteroid and firmicutes phyla may become dominant for the rest of their lives (82–84).

## Influence of the Mediterranean diet on breastfeeding

Since there is a direct correlation between the quality of nutrition and the growth of the fetus, feeding during the periconceptional phase and breastfeeding is one of the stages of feeding that has been the subject of much scientific research. Current research demonstrates how specific dietary habits can help prevent and treat the clinical issues that expectant moms may develop during their pregnancy. Considering this, the Mediterranean diet is emphasized as being essential for the management and treatment of these issues, such as preeclampsia and gestational diabetes mellitus, which can result in intrauterine growth retardation, neural tube defects, cardiovascular, and metabolic diseases that can persist into adulthood (85, 86).

The Mediterranean diet is known for maintaining high consumption patterns of fruits, vegetables, legumes, whole grains, and healthy fats, these food groups provide an environment conducive to the colonization of beneficial bacteria in the intestinal microbiota, including in infants (87–90). Although most of the studies that demonstrate the influence of this diet have been carried out in the adult population, they could be extrapolated to the infant population, because they are food groups that can and should be introduced in the first years of life within their complementary feeding plan (90). Due to the nutrients provided by this diet such as fiber, healthy fats, antioxidants, and polyphenols contribute to creating a favorable environment for the modulation of the intestinal microbiota (91). So, the Mediterranean diet pattern is centered on including food groups in sufficient amounts and proportions, it can be feasible to meet the increased nutritional needs during this period. This diet’s hallmark is its continued emphasis on fruits and vegetables at the expense of minimal animal-based fats and proteins. According to the WHO, this must be combined with physical activity and refraining from alcohol and tobacco use (85).

Breastfeeding is the only and primary source of nutrition for the first six months of life, so it plays a crucial function beyond the gestation period. It is important to emphasize that breast milk must provide the baby with the best nutrition possible and be suitable in its composition, considering the baby’s regular diet’s supply of macro and micronutrients.

Because vegetables comprise most of the diet, the Mediterranean offers a high energy density. The source of high energy density in the Mediterranean diet can be provided by starchy vegetables, which have a higher content of complex carbohydrates (92). In them, we can include potatoes, corn, sweet potatoes, and peas, these are a source of immediate and sustained energy; they are satiating; because they maintain the feeling of satiety and consequently are beneficial for weight control. Additionally, (86) states that the amount of essential fatty acids in breast milk is directly related to the amount consumed through diet, with olive oil serving as the primary source of lipids. Improving the composition of breast milk can enhance the endogenous synthesis of DHA, which in turn can enhance children’s development of their visual and cognitive faculties. On the other hand, young children can develop to their ideal weight and height if a source of high-quality food categories is available in sufficient amounts, as established. Establishing dietary patterns based on the Mediterranean diet does not influence the overall composition of breast milk, since it is a high-quality food, regardless of the diet of the mother (93). Breast milk is adapted to the needs of the newborn, providing the macronutrients necessary for its development (94, 95).

However, it is worth mentioning that maintaining a balanced diet provides benefits in certain components such as increasing the quality of nutrients and the percentage of omega 3. Several studies show that a diet based mainly on fruits and vegetables and healthy fatty acids can modify the composition of only certain nutrients. In the case of pregnant mothers who have a higher intake of omega-3 may have higher levels of DHA in their milk, providing benefits to brain and cardiovascular health, as well as improving the composition of the intestinal microbiota (96).

The consumption of fats in this type of diet is minimal, but the main consumption of healthy fats such as olive oil, composed of monounsaturated fats that promote the increase of HDL cholesterol, is also rich in antioxidants, such as polyphenols that generate anti-inflammatory properties, due to its content of DHA (Docosahexaenoic Acid) (97, 98). Consuming foods rich in DHA modulates the intestinal microbiota because it favors the growth of beneficial bacteria such as lactobacillus and bifidobacterium, since Omega 3 is partially metabolized by these bacteria, thus improving the distribution of the intestinal flora and preventing the dysregulation of the microbiota (98, 99).

## Discussion and conclusion

According to the analysis, maintaining a balanced diet not only provides the vitamins and minerals necessary for the performance of the body’s main functions but also provides macronutrients such as protein, carbohydrates, healthy fats, fiber, and water. All are provided in a balanced way, both macro and micronutrients, ensure recovery in the postpartum period, provide the benefit of supply of nutrients needed in breast milk for the development of the pregnant woman, and reduce the risk of long-term nutritional deficiencies in both the infant and the mother. So, the periconceptional stage is a unique time that preserves a close connection with genetic programming and, thus, has the potential to significantly impact elements like early health, risk of developing obesity, and complications in adult life. It’s important to emphasize that specific epigenetic modifications can affect gene expression in metabolism, fat regulation, and appetite; this could be attributed to other environmental influences during this time as well as parental nutrition, particularly maternal nutrition. As a result, during the first 1,000 days of life, breastfeeding becomes one of the most crucial strategies to lower the risk of childhood obesity.

Exclusive breastfeeding can influence DNA methylation and gene expression related to metabolism and the immune system, potentially reducing the risk of obesity, metabolic disorders, and other conditions later in life. A multifactorial and multidisciplinary approach is needed to combat childhood obesity, addressing biological, environmental, social, and behavioral factors early on. This includes promoting a balanced diet for the mother to ensure adequate intake of vitamins and minerals. It also includes promoting exclusive breastfeeding and appropriate transition to complementary feeding to ensure healthy child and family nutrition. In addition, interventions targeting the various determinants of obesity should be implemented.

Within these actions, dietary patterns are considered fundamental, with the Mediterranean diet being a recommended option for pregnant and lactating women. This diet, rich in fruits, vegetables, and olive oil, is considered nutritionally complete. Characterized by its caloric density, it can help meet the increased nutritional needs during this period, thus preventing complications such as pre-eclampsia, gestational diabetes, inadequate intrauterine growth, and neural tube defects. In turn, it can potentially improve the composition of breast milk, which benefits infant development. The Mediterranean diet is a recommended option for pregnant and lactating women, due to the multiple benefits it can bring to the health of individuals. However, it is not the only option, there are a variety of diets that can be established during this period, according to the conditions or nutritional needs and food preferences of patients. It should be emphasized that the common denominator of the diet that is applied should provide all macro and micronutrients in a balanced and balanced way, prioritizing quality foods that can benefit health.

All these interventions mentioned above, in a multifactorial manner, considering all aspects of preventing overweight and obesity, would be effective if they were carried out in a preventive manner. It is essential to know how inadequate nutrition during the periconceptional period of the mother can have repercussions on the life of the children, even from their intrauterine stage, with epigenetic changes that can contribute to the development of chronic non-communicable diseases that affect them throughout their life stages.

Working in a comprehensive and interdisciplinary manner, health professionals can identify risk factors at these stages and link them to scientific and medical research. It is recommended that further research be conducted in areas such as epigenetics, breastfeeding, and childhood obesity to find relevant findings that will allow us to intervene in the nutritional status of individuals and, in turn, prevent future health complications. Research and scientific dissemination in these areas are essential to better understand the underlying mechanisms and develop effective early intervention strategies. Through a multidisciplinary and evidence-based approach, we can comprehensively address the challenges related to nutrition, breastfeeding, and obesity prevention in children, thus promoting healthy development from the earliest stages of life.

## Data Availability

The original contributions presented in the study are included in the article/supplementary material, further inquiries can be directed to the corresponding authors.
